# E-Cigarette Advertising in the UK: A Content Analysis of Traditional and Social Media Advertising to Observe Compliance with Current Regulations

**DOI:** 10.1093/ntr/ntab075

**Published:** 2021-04-15

**Authors:** Martine Stead, Allison Ford, Kathryn Angus, Anne Marie MacKintosh, Richard Purves, Danielle Mitchell

**Affiliations:** Institute for Social Marketing and Health, University of Stirling, Stirling, UK

## Abstract

**Introduction:**

The advertising of e-cigarettes in the UK is regulated through the revised EU Tobacco Products Directive and the Tobacco and Related Products Regulations, with further rules set out in the Advertising Standards Authority (ASA) Committees of Advertising (CAP) Code. Focusing on the ASA CAP Code Rules, we examined e-cigarette advertising regulation compliance in traditional advertising channels and on social media.

**Methods:**

We conducted a content analysis of UK e-cigarette and related product advertising using a randomly selected sample (*n* = 130) of advertising in traditional channels and on Instagram which appeared between January and December 2019. All ads were independently double-coded to assess compliance with each CAP Code Rule.

**Results:**

In traditional channels, our sample of advertising had largely good compliance. Only very small numbers of these ads appeared to be clearly in breach of any of the ASA rules (5% were in breach of Rule 22.7; 2% of Rule 22.9; and 1% of Rule 22.10). In contrast, we judged that all of the Instagram sample (*n* = 30) was in breach of Rule 22.12. For some rules, it was not possible to make definitive judgments about compliance, given uncertainty regarding how a rule should be interpreted and applied.

**Conclusions:**

We found overall good compliance for advertising in traditional channels, but assessed all of our social media advertising samples was in breach of regulations. Current guidance on e-cigarette advertising could be improved to facilitate e-cigarette advertising assessment and regulation. It would be beneficial to bring consumer perspectives into the assessment of regulation compliance.

**Implications:**

The regulation of e-cigarette advertising is a global concern. The UK Government has a statutory obligation to review the Tobacco and Related Products Regulations by May 2021. This study assessed compliance with current UK e-cigarette advertising regulations on placement and content. We identified areas where greater clarity is needed and outlined implications for future regulation.

## Introduction

There is an ongoing debate over how best to regulate e-cigarette advertising to ensure that it does not renormalize smoking or attract non-smokers and non-nicotine users.^1^ It is also important that regulations keep pace with developments in marketing communications, particularly in relation to social media which has seen an increase in marketing activity for e-cigarettes in recent years.^2–4^

**Figure 1.**
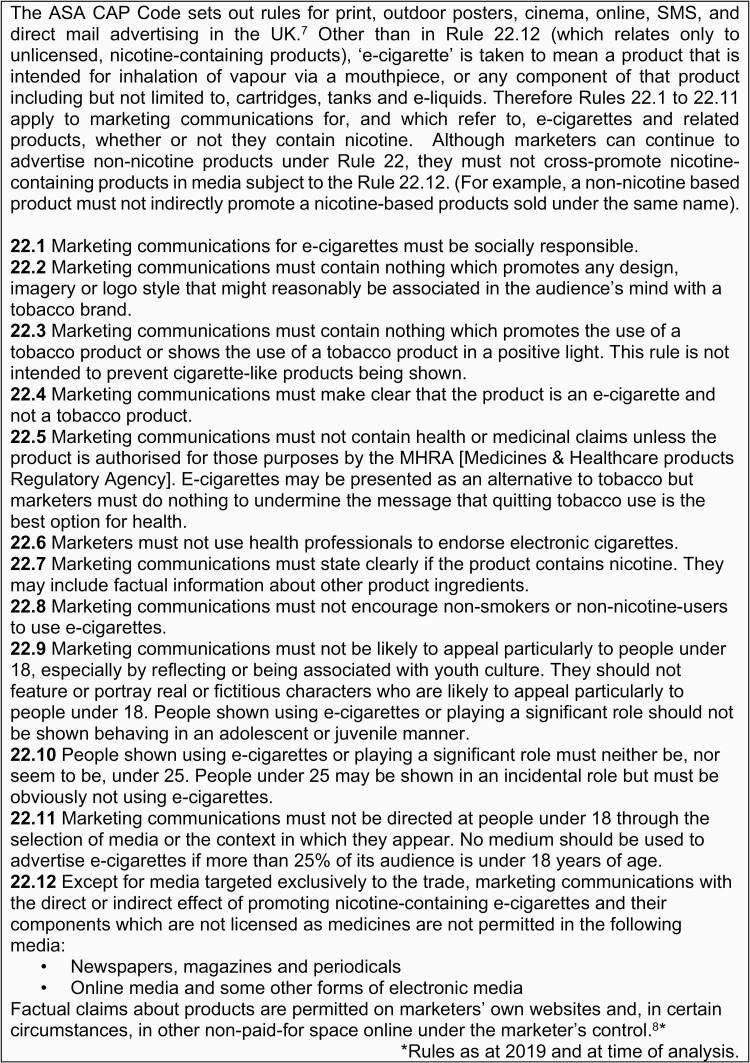
UK Code of Non-broadcast advertising and direct & promotional marketing (Committee of Advertising Practice Code) Rule 22.

The advertising of e-cigarettes in the UK is regulated through Article 20(5) of the revised EU Tobacco Products Directive (2014/40/EU) (TPD),^5^ which was transposed into UK law by the Tobacco and Related Products Regulations (TRPR) 2016. The TPD prohibited the advertising of nicotine-containing e-cigarettes (unless licensed as medicines) in channels with potential cross-border impact (i.e. channels that show adverts or sponsored events that originate from non-EU countries in EU countries), including TV, radio, newspapers, magazines, and sponsorship. Online advertising was also prohibited, although the regulations left scope for marketers to retain websites containing factual information about e-cigarette products. In its application of the TPD and the new TRPR, the UK Government aimed to achieve a balance between encouraging current smokers to switch from tobacco to e-cigarettes, and protecting never smokers, particularly children, from viewing the products as appealing.^6^ As there are currently no medicinally licensed nicotine vaping products in the UK, the prohibitions apply to all nicotine-containing e-cigarette products on the market. In the UK, the TRPR requirements for e-cigarette advertising were set out in 2017 and are enforced by the Committee of Advertising Practice (CAP) – a self-regulatory body of organizations representing advertising, direct marketing, media businesses, and sales promotion endorsed and administered by the independent Advertising Standards Authority (ASA).^7^ These rules appear in section 22 of the ASA CAP Code (Figure 1).^8^

Although Rule 22.12 prohibits advertising in online media, social media content for e-cigarettes is permitted in “non-paid-for space online under the marketer’s control” providing that the content is “factual” rather than “promotional.” ^9^ Promotional content is deemed to include health claims, descriptive language or significant imagery unrelated to the product, that goes beyond objective facts. Social media, which are particularly popular among adolescents and young adults,^10,11^ present an ideal platform for e-cigarette brands and specialist e-cigarette retailers to promote their products using aesthetically appealing imagery and videos.^6,12,13^

Our study, therefore, examined e-cigarette and related product advertising in both traditional advertising channels and on social media. For the purpose of this paper, we use the term “traditional” to refer to paid-for channels in which e-cigarette advertising occurs in the UK (e.g. outdoor, cinema, direct mail). We assessed whether e-cigarette advertising complies with existing regulations by focusing on the ASA CAP rules, and considered implications for future regulation. The UK Government has a statutory obligation to review the TRPR by May 2021.^14^ Although this study was conducted to inform the UK review, the findings have wider relevance for other jurisdictions currently debating the appropriate regulation of e-cigarette advertising, both in the EU and elsewhere.

## Methods

We conducted an in-depth content analysis of UK e-cigarette advertising. Content analysis is an established method for identifying, describing, and quantifying the different elements of advertising^15–17^ and for assessing advertising regulation compliance.^18–20^

### Sample Selection

Two samples of advertising were selected (Table 1). Firstly, we purchased all UK advertising “creatives” (real-world advertising examples) for e-cigarette products, brands, and retailers captured by the media agency Nielsen in 2019 (*n* = 134).^21^ Nielsen captured e-cigarette advertising in the following channels: cinema, direct mail, door drops (print leaflets delivered to the home without a specified addressee), internet, outdoor, and press. Nielsen’s proprietary media monitoring method also covers television, radio, and email, however, no e-cigarette ads were captured in these channels. A simple random sample of 100 was selected for analysis. Secondly, we selected Instagram posts from the official accounts of three popular brands: blu (Imperial Tobacco) and Logic Vapes (JTI), the second and third highest selling brands on the UK convenience market in 2019,^22^ and Totally Wicked (non-tobacco company-owned),^23^ a popular online and high street retailer.^24^ Instagram was chosen to represent advertising content through social media as it is one of the most popular social media platforms in the UK,^25^ particularly among adolescents and younger adults^10,11^ who are a longstanding target market for the tobacco industry. For each brand, we collected all posts between 1st January and 31st December 2019 using screenshots and screen recording. For blu and Logic, a researcher had to request to “follow” the accounts, which were private, while Totally Wicked’s posts were publicly accessible. From a total of 405 posts, a simple random sample of 10 posts was selected for each brand (*n*= 30: 26 single image and four videos). The sample sizes (100 creatives and 30 Instagram posts) were determined by resource constraints. We use the term “ad” to refer to the sample of both creatives and Instagram posts. Seventy-five percent of the sample of ads were from tobacco company-owned brands, and 25% from non-tobacco company-owned brands.

**Table 1.** Sample of UK e-cigarette advertising (1st January-31st December 2019)

Media channel	n	%
Advertising creatives (from Nielsen):		
Cinema	4	3
Direct mail	4	3
Door drops	8	6
Internet	5	4
Outdoor	66	51
Press	13	10
Social media sample:		
Instagram posts by three brands	30	23
**Total**	**130**	**100**
Brand owner:		
Tobacco company owned brand	97	75
Non-tobacco company-owned brand	33	25

### Codebook Development

We developed an initial codebook, informed by previous content analysis studies of e-cigarette advertising,^26–28^ and of gambling marketing and advertising compliance with ASA codes,^20,29,30^ and by examining a selection of ads not included in the final sample. Using SPSS (version 25), we piloted the codebook on a random 10 ads from our full sample. Following team discussion, the codebook was refined by adding or removing codes, re-ordering items, clarifying descriptions, and adding freetext response options. A second test using a different random 10 ads from the full sample was then conducted, after which the codebook was finalized.

### Measures

Measures to assess compliance with e-cigarette advertising regulations were derived from Rules 22.2 to 22.11 of the CAP Code (Figure 1). For each rule, we assessed whether ads complied using Yes/No/Not sure response options. Because of the subjective nature of this assessment, a “Yes” response indicated *reasonable evidence* that the ad contained content that could be deemed in breach of the CAP Code rule (or a “No” response where the CAP Code rule was expressed as a positive requirement). We did not code compliance with Rule 22.1, e-cigarette marketing communications “*must be socially responsible*,” as we did not view this as a standalone code, but one which linked to Rules 22.8 “*ads should not encourage non-smokers or non-nicotine users to use e-cigarettes*,” 22.9 “*likely to appeal particularly to people under 18, especially by reflecting or being associated with youth culture,”* and 22.10 “*does the ad show people using e-cigarettes or playing a significant role who are, or seem to be, under 25?”* Any ads viewed in breach of these rules would automatically be viewed in breach of Rule 22.1 and socially irresponsible.

We also assessed ads in relation to Rule 22.12 (Figure 1). This was a more complex process requiring a two-stage assessment: firstly, the media channel used, and, for Instagram ads only, an assessment of whether ads contained factual versus promotional claims, based on ASA guidance.^9^ The ad was judged promotional if it contained any promotional language or imagery which went beyond purely factual content, for example, promotional descriptions of flavors of products, or imagery that evoked a particular lifestyle or humor.

### Coding Process

For each measure, each ad was independently coded by two researchers: KA coded all ads, and AF, DM, and RP each coded a randomly allocated third. The independent double-coding process^31,32^ reduces the possibility that the response is influenced by a single researcher’s biases, reduces the risk of coding errors, and meant that intercoder reliability calculations were not required. On coding completion, KA, MS, AMM, and AF discussed and resolved any coding divergences for each item. Coding results raised queries about CAP Code Rule 22, and we sought advice from the ASA for help with interpretation via one conference call.

### Analysis

Data were analyzed using SPSS (version 25). Descriptive statistics were computed for compliance with the individual CAP Code Rules. Bivariate analysis, using the Chi-square test, was conducted to compare the proportion of ads complying with each CAP Code by whether the ad was for a tobacco company-owned brand or a non-tobacco company-owned brand. Due to the small expected cell size for CAP Code Rules 22.9 and 22.10, the Fisher’s Exact Test statistic was used.

## Results

The results are presented around the individual CAP Code Rules 22.2 to 22.12.

### Rules 22.2 – 22.4. Association with Tobacco, Promotion of Tobacco or Confusion with Tobacco

All ads appeared to comply with CAP Code Rules 22.2 to 22.4 (Table 2). We judged that no ads contained imagery that might be associated with a tobacco brand (Rule 22.2). Although some e-cigarette packaging featured in the ads appeared to resemble branded tobacco packaging, we assessed that this did not bring to mind specific tobacco brands, nor did it resemble current standardized tobacco packaging. We judged that no ads promoted a tobacco product or showed the use of tobacco products in a positive light (Rule 22.3). We did not observe any ads which might confuse e-cigarettes with a tobacco product (Rule 22.4). Some ads were potentially ambiguous in terms of what was actually being advertised – for example, ads that did not specifically show an e-cigarette product – but we judged that they did not cause any confusion that the ad was for a tobacco product.

**Table 2.** Compliance with CAP Code Rule 22

Variable	Yes %	No %	Not sure %	Chi-square *p* Value^*^
Rule 22.2 Does the ad promote any design, imagery or logo style that might reasonably be associated in the audience’s mind with a tobacco brand?	0	100	0	
* Tobacco-company owned brand ads:*	*0*	*100*	*0*	
* Non-tobacco-company owned brand ads:*	*0*	*100*	*0*	*n.s.*
Rule 22.3 Does the ad contain anything which promotes the use of a tobacco product or shows the use of a tobacco product in a positive light?	0	100	0	
* Tobacco-company owned brand ads:*	*0*	*100*	*0*	
* Non-tobacco-company owned brand ads:*	*0*	*100*	*0*	*n.s.*
Rule 22.4 Does the ad make it clear that the product is an e-cigarette and not a tobacco product?	100	0	0	
* Tobacco-company owned brand ads:*	*100*	*0*	*0*	
* Non-tobacco-company owned brand ads:*	*100*	*0*	*0*	*n.s.*
Rule 22.5 Does the ad contain medicinal claims unless the product is authorized for those purposes by the MHRA?	0	94	6	
* Tobacco-company owned brand ads:*	*0*	*94*	*6*	
* Non-tobacco-company owned brand ads:*	*0*	*94*	*6*	*n.s.*
Rule 22.6 Does the ad use health professionals to endorse electronic cigarettes?	0	100	0	
* Tobacco-company owned brand ads:*	*0*	*100*	*0*	
* Non-tobacco-company owned brand ads:*	*0*	*100*	*0*	*n.s.*
Rule 22.7 Does the ad clearly state if the product contains nicotine?	76	24	0	
* Tobacco-company owned brand ads:*	*89*	*11*	*0*	
* Non-tobacco-company owned brand ads:*	*39*	*61*	*0*	*p<0.001*
Rule 22.8 Does the ad contain any content which might encourage non-smokers or non-nicotine-users to use e-cigarettes?	0	75	25	
* Tobacco-company owned brand ads:*	*0*	*75*	*25*	
* Non-tobacco-company owned brand ads:*	*0*	*73*	*27*	*n.s.*
Rule 22.9 Is the ad likely to appeal particularly to people under 18, especially by reflecting or being associated with youth culture?	2	92	5	
* Tobacco-company owned brand ads:*	*1*	*99*	*0*	
* Non-tobacco-company owned brand ads:*	*6*	*73*	*21*	*n.s.* ^**^
Rule 22.10 Does the ad show people using e-cigarettes or playing a significant role who are, or seem to be, under 25?	1	65	34	
* Tobacco-company owned brand ads:*	*0*	*59*	*41*	
* Non-tobacco-company owned brand ads:*	*3*	*85*	*12*	*n.s.* ^**^
Rule 22.11 Is the ad directed at people under 18 through the selection of media or the context in which they appear?	0	38	62^#^	
* Tobacco-company owned brand ads:*	*0*	*39*	*61* ^#^	
* Non-tobacco-company owned brand ads:*	*0*	*33*	*67* ^#^	*n.s.*
Rule 22.12 Is the ad in a permitted channel?	63	37	0	
* Tobacco-company owned brand ads:*	*68*	*32*	*0*	
* Non-tobacco-company owned brand ads:*	*48*	*52*	*0*	*p = 0.044*

### Rules 22.5 – 22.6. Medicinal Claims and Health Professional Endorsement

The vast majority of ads (94%) complied with Rule 22.5 and did not contain medicinal claims (Table 2). We coded eight ads (6%) as “not sure” as we considered it plausible that they created an implicit association between quitting smoking and the brand or product advertised (Table 3). All ads appeared to comply with Rule 22.6 which prohibits the use of health professionals to endorse e-cigarettes.

**Table 3.** Examples of e-cigarette ads coded as “in breach” or “not sure”

Variable (Rule)	Examples of ads assessed as “in breach” or “not sure”
22.5 Does the ad contain medicinal claims unless the product is authorized for those purposes by the MHRA?	**Not sure** examples (6% of ads): judged as implying products aided cessation: • cinema ad featuring testimonial “I would never go back to smoking” • Totally Wicked Instagram ads: #Quitforlife, #SmokingCessation hashtags • Juul internet ad: “The average smoker tries to quit 30 times. Make the switch” • Juul direct mail ad: “To impact the lives of the world’s 1 billion adult smokers and ultimately eliminate cigarettes.”
22.7 Does the ad clearly state if the product contains nicotine?	**Clearly in breach** examples (5% of ads): • ads showing nicotine-containing products such as e-liquid and pod refills • Nic-Hit Pro device ad (can only be used with nicotine-containing products). **Non-compliant** examples (19% of ads): • ads referring to specific devices (e.g. MyBlu and Totally Wicked Orbis/Arc Instagram ads), or e-liquid shortfills (e.g. Ruthless shortfills), to which nicotine may or may not be added depending on consumer preference • ads which *indirectly* promoted nicotine-containing product ranges: e.g. a Totally Wicked Instagram ad linking the brand with a children’s charity; Juul ad advocating a rise in the age of sale; blu Instagram ad showing a female exhaling vapor; ads referring to a retailer (e.g. VPZ, PRO VAPE) but no specific products.
22.8 Does the ad contain any content which might encourage non-smokers or non-nicotine-users to use e-cigarettes?	**Not sure** examples (25% of ads): judged as including messages or imagery that may have broad appeal and no “for adult smokers/vapers” message: • ads promoting “starter kits,” a term which may speak to new users • five VPZ ads making references to football • internet ad promoting free samples • a Diamond Mist ad featuring a female face with “Claire’s crazy for cola” text.
22.9 Is the ad likely to appeal particularly to people under 18, especially by reflecting or being associated with youth culture?	**In breach** examples (2% of ads): • Totally Wicked Instagram ad: man in fancy-dress and child’s birthday cake • blu Instagram ad: “flavor rooms” featured, resembling children’s soft play areas and filled with plastic balls, inflatable fruits, and space hoppers • VPZ press ad showing a young Scottish footballer, then aged 20 yrs. **Not sure** examples (5% of ads): • cartoons (e.g. Totally Wicked Mr Wicked devil, Pro Vape political caricatures) • Totally Wicked Instagram ad: youthful feminine imagery (love hearts, flowers, butterflies) and #girlswhovape hashtag • VPZ press ad associating e-cigarettes with football.
22.10 Does the ad show people using e-cigarettes or playing a significant role who are, or seem to be, under 25?	**In breach** examples (1% of ads): • VPZ press ad depicting 20-year-old footballer. **Not sure** examples (34% of ads): • could not infer character ages, plausible they could be either side of 25 yrs • could not clearly see face(s) or only a hand or arm shown • graphic illustration style used (e.g. blu outdoor ads) which made it difficult to infer the ages of people depicted.
22.11 Is the ad directed at people under 18 through the selection of media or the context in which they appear?	**Insufficient information to classify** examples (62% of ads): • no data indicating where many outdoor ads were positioned • internet ads as we had no data indicating where they appeared • cinema ads as we had no data on which films were played after the ads • all Totally Wicked Instagram ads appearing on a public account, for which any hashtags can appear publicly on the corresponding hashtag page.
22.12 Is the ad in a permitted channel?	**In breach** examples (37% of ads): • all press ads: VPZ press ads and Logic press inserts • all internet ads for Juul and PRO VAPE • all Totally Wicked Instagram ads as they originated from a public account • all Instagram ads: all Totally Wicked, blu, and Logic’s posts were judged to contain promotional content.

### Rule 22.7. Clear Statement on Nicotine Content

The majority of ads (76%) stated that the product contains nicotine (Table 2). The majority of ads from tobacco-company owned brands included a nicotine statement while only a minority of the ads from non-tobacco-company owned brands included a statement (89% v. 39%, *p* < 0.001). Around a quarter (24%, *n* = 31) did not contain a statement on nicotine content, including all Instagram ads by two brands (blu and Totally Wicked) and all press ads by retailer VPZ. Six of the ads (5% of the overall sample) without nicotine content statements were for nicotine-containing products and in our view should clearly have included a statement. The remainder were of two kinds: ads for devices or e-liquid shortfills, to which nicotine may or may not be added depending on consumer preference (*n* = 13), and ads which did not *directly* refer to a specific e-cigarette product, but which indirectly promoted product ranges with nicotine-containing products through the use of an identical brand or retailer name (*n* = 11) (Table 3). It is currently unclear in the CAP Code and associated guidance whether these two kinds of ads should include a statement on nicotine content. A further one ad showed an e-liquid range, but it was difficult to tell from the image whether the specific products shown contained nicotine; the range includes a variety of nicotine strengths including a nicotine-free option. Overall, therefore, we assessed that while 24% of the ads did not contain a statement on nicotine content, 5% were unequivocally in breach of the rule, with uncertainty regarding whether the remaining 19% should be deemed in breach or not.

### Rule 22.8. Encouragement of Non-smokers or Non-nicotine Users

We assessed that three-quarters of ads (75%) did not contain “*any content which might encourage non-smokers or non-nicotine-users to use e-cigarettes*” (Table 2). We judged that ads that were product-focused, using technical language such as “maximum nicotine, acts faster, lasts longer” and “hyper-real vapor,” would likely speak to those already using e-cigarettes. We also judged that all Instagram ads from a private account would not encourage non-smokers or non-nicotine-users, since content from private accounts cannot be accessed by or pushed to non-subscribers. While we did not identify any ads as being in breach of this rule, we did categorize 25% as “not sure.” These were ads with messages or imagery which we considered might appeal to a broad range of people and which did not contain a statement that the product was “for adult smokers/vapers” (see Table 3 for examples).

### Rules 22.9 – 22.11. Appeal to Under 18s, Depiction of People Under 25 and Directed at Under 18s Through Media Placement

We assessed that the majority of ads (92%) were unlikely to appeal to people under 18 (Rule 22.9; Table 2). Three ads (2%) were identified as plausibly appealing to people under 18 due to their clear association with youth culture or feature of a celebrity with likely youth appeal. A further seven ads (5%) were coded as “not sure,” exclusively from non-tobacco-company owned brands (see Table 3 for examples).

We judged that the majority of ads (65%) did not appear to “*show people using e-cigarettes or playing a significant role who are or seem to be, under 25*” (Rule 22.10). However, 34% were coded as “not sure,” including both tobacco-company owned and non-tobacco-company owned brands (see Table 3). Only one ad (1%), depicting a footballer who was 20 years at the time of the ad, was assessed as being in breach of the Code.

Rule 22.11 refers to the placement of the ad: the publication title, context or location in which it was originally placed. We judged that 38% of ads were not directed at people under 18 “*through the selection of media or the context in which they appear*.” These were all the direct mail and door drop ads, the 20 Instagram ads which appeared on private accounts, and 19 of the outdoor ads where Nielsen data indicated that the audience was likely to be a general public one (i.e. to comprise no more than 25% under 18s); these were primarily ads placed on transport or located in identifiable shopping centers. For the remaining 62% of ads we did not have sufficient placement information to assess potential audience composition and therefore coded them as “insufficient information to classify” (Table 3).

### Rule 22.12. Placement in Permitted and Non-permitted Media Channels

Sixty-three percent of our sample had been placed in permitted channels (cinema, direct mail, door drops and outdoor; Table 2). Fourteen percent (*n* = 18) were judged, on the basis of their categorisation by Nielsen, to have been placed in prohibited channels: internet (five ads, 4%) and press (13 ads, 10%). Of the press ads, eight were described by Nielsen as inserts within magazines or newspapers. It is unclear whether the prohibition on press advertising extends to inserts. The remaining five press ads did not *directly* promote nicotine-containing e-cigarettes by name or imagery; however, as the retailer name (VPZ) is also the name of an e-cigarette product range, we judged that these ads *indirectly* promoted nicotine-containing e-cigarette products and therefore were in breach of this rule.

The remaining 23% of the sample comprised Instagram ads. ASA guidance states that advertising is permitted in “non-paid-for space online under the marketer’s control” providing the content is “factual” rather than “promotional.” ^9^ We judged Instagram as “*non-paid-for space online under the marketer’s control*” when accounts were private, where content could only be found by subscribers. Content on public accounts can be accessed by or pushed to those not actively seeking it by appearing in the Search and Explore section or on a corresponding hashtag page. As the Totally Wicked account was public, we judged that these Instagram ads (*n* = 10) were not in a space under the marketer’s control and were in breach of this rule. We then assessed whether the content of the Instagram ads was factual or promotional.^9^ We judged that all Instagram ads contained promotional language or imagery which went beyond the objective, factual content: for example, lifestyle or humorous imagery, the descriptive promotional language around flavors (“fresh”), or product design (“slick”), and the use of hashtags such as #vapelife, #vapefam, and #vapeporn. We judged that such content went beyond factual and that the ads were therefore in breach of the rule, even though some of the ads appeared on private accounts.

Ads for non-tobacco company-owned brands were more likely than tobacco company-owned brands to be in non-permitted media channels (52% v. 32%, *p* < 0.05).

## Discussion

Our findings paint a contrasting picture with regard to e-cigarette advertising in traditional channels and in social media in the UK. We assessed that all of the ads in the Instagram sample were in breach of ASA CAP Code Rule 22.12, which only permits advertising in online media where the online space is “under the marketer’s control” and where the content is “factual” rather than “promotional.” In traditional channels, our sample of cinema, direct mail, door drops, internet, outdoor and press, had largely good compliance. We found no ads which promoted the use of tobacco products or showed the use of tobacco in a positive light, no ads which suggested confusion between e-cigarettes or tobacco, and no ads which used health professionals to endorse e-cigarettes.

We judged only a very small number of ads to be in breach of any of the ASA rules. These related to Rule 22.9, which concerns appeal to people under 18 (2%), Rule 22.10, which concerns the depiction of people who are or seem to be under 25 (1%), and the 5% of ads which we judged to be in breach of Rule 22.7, which requires ads to include a statement on nicotine content. However, for several rules, we coded some ads as “not sure.” This judgment often represented uncertainty regarding how a rule should be interpreted and applied, and in some cases, lack of information to make a full assessment. Where there was non-compliance or uncertainty, this was generally found across ads from both tobacco and non-tobacco company-owned brands, with no significant differences. The exception was Rule 22.7, where not including a statement on nicotine content was significantly associated with an ad from a non-tobacco company-owned brand. That compliance with this Rule was mainly from tobacco company-owned brands, suggests that tobacco companies are paying more attention to this requirement, and conversely that non-tobacco company-owned brands may be less aware of the importance of Rule 22.7.

### Implications for E-Cigarette Advertising Regulation

Consistent with previous studies,^2,4,12,13,28,33,34^ our study identifies social media advertising as a focus of concern. The two-stage process we had to follow to assess whether the Instagram ads complied with Rule 22.12 on media placement – firstly, assessing whether they were in an online space under the marketer’s control and secondly whether the content was factual or promotional – illustrates that the guidance is not easy to interpret or apply. Greater clarity is needed, for each social media platform and other types of online space, on what would constitute being under the marketer’s control (for example, is it sufficient that a space can only be accessed by subscribers, given that age-verification processes can be easily circumvented?).^35^ This ambiguity may account for the amount of Instagram content we judged to be in breach of ASA Rules. Recently, social media platforms themselves have announced voluntary actions to limit e-cigarette advertising. In December 2019, Instagram announced a ban on “influencers” promoting vaping products and Facebook stated that it no longer allowed adverts for the sale or use of electronic cigarettes.^36,37^ Further research should monitor these and other voluntary initiatives to assess whether they are sufficient or whether mandatory restrictions are required.^33^

More specific guidance for advertisers and regulators would be beneficial in other areas. Rule 22.7 requires e-cigarette ads to state if the product contains nicotine. Clarity is needed on whether a nicotine statement is required for ads that do not directly refer to a specific product but which indirectly promote product ranges with nicotine-containing products through the use of an identical brand or retailer name, as was the case with 19% of the ads in our sample which did not contain a nicotine statement. It could be argued that ads which indirectly promote nicotine-containing products should be subject to the rules.

Our study demonstrates the challenges in assessing potential appeal to under 18s or non-smokers/non-nicotine users, and whether people in ads appear to be under 25. We could not assess the age of people shown in around a third of ads because they were depicted as graphic illustrations rather than photos, they appeared either side of 25 years, or only a body part was shown. Clearer guidance would assist regulators in assessing age in these instances. Detailed consumer research – for example, qualitative research with young people, non-smokers, or non-nicotine users – could provide valuable evidence to regulators on the potential appeal and persuasive effect of ads. This approach has previously been used to assess compliance with alcohol advertising rules in the UK and Australia.^38,39^ While ad effects on e-cigarette ad and product appeal, and intention to try have been explored previously,^12,13,40–45^ much of this work comes from North America. There is a need for up-to-date, country-specific research given that e-cigarette markets, marketing, and policy environments vary greatly between countries and over time. Findings produced elsewhere may lack relevance or be interpreted differently. It is also important that future studies explore current tobacco and/or nicotine users’ interpretation of e-cigarette advertising and whether it is able to provide messages which may help to promote e-cigarettes as an alternative to smoking while abiding by current regulations to protect non-users.

### Strengths and Limitations

To our knowledge, this is the first assessment of whether e-cigarette advertising in the UK complies with current regulations. Our detailed codebook was robustly piloted, and our independent double-coding method increased accuracy and reduced bias. Ads were randomly selected, and therefore not influenced by seasonality. In terms of limitations, assessments of characteristics such as advertising appeal to youth by adult researchers are necessarily subjective. It is possible that our interpretation of the ads may differ from that of young people. Therefore, as we noted above, it would be beneficial for young people to be involved in the assessment of such advertising. Nielsen did not include point-of-sale monitoring, meaning that point-of-sale advertising, a key marketing tool,^46^ was omitted from our analysis, and although extensive, it is not certain that all relevant ads in monitored channels were detected through Nielsen’s proprietary media monitoring. Our social media sample was restricted to Instagram; practices may differ on other platforms Finally, our sample of ads was restricted to 2019, and subsequent developments such as Instagram’s ban on influencers from promoting vaping products may have affected e-cigarette advertising practice.^37,47^ In addition, Covid-19 is likely to have affected e-cigarette advertising volume, channel selection, and creative strategies.

## Conclusions

Our study provides a rich and detailed assessment of compliance with current UK e-cigarette advertising regulations. We found overall good compliance for advertising in traditional channels but assessed that all of the social media advertising in our sample was in breach of regulations. We also identified several areas where current guidance could be improved to facilitate e-cigarette advertising assessment and regulation, and highlight the importance of bringing consumer perspectives to bear on this process.

## Supplementary Material

A Contributorship Form detailing each author’s specific involvement with this content, as well as any supplementary data, are available online at https://academic.oup.com/ntr.

* Chi-square tests of difference in the proportion of “yes” responses by whether the brand was a tobacco company-owned brand or not. ^**^ Based on Fisher’s exact test due to small expected cell size. ^#^ Insufficient information to classify due to lack of information on location/context of ads.

ntab075_suppl_Supplementary_Taxonomy_Form
